# Educational Case: Hairy cell leukemia

**DOI:** 10.1016/j.acpath.2025.100180

**Published:** 2025-05-19

**Authors:** Cassandra M. Raziel, Joanna L. Conant

**Affiliations:** a Larner College of Medicine, University of Vermont, Burlington, VT, USA; b Department of Pathology and Laboratory Medicine, University of Vermont Medical Center, Burlington, VT, USA

**Keywords:** Pathology competencies, Diagnostic medicine, Hematology, Diagnosis of white cell disorders, Genetic mutations in hematologic malignancy, Targeted therapy, Hairy cell leukemia

## Primary objective

Objective H5.1: WBC Count and Differential. Describe, with examples, how abnormalities of WBC count and WBC differential count can be used along with patient history to direct laboratory testing in the workup of a patient.

Competency 3: Diagnostic Medicine and Therapeutic Pathology; Topic: Hematology (H); Learning Goal 5: Diagnosis of White Cell Disorders.

The following fictional case is intended as a learning tool within the Pathology Competencies for Medical Education (PCME), a set of national standards for teaching pathology. These are divided into three basic competencies: Disease Mechanisms and Processes, Organ SystemPathology, and Diagnostic Medicine and Therapeutic Pathology. For additional information, and a full list of learning objectives for all three competencies, see https://www.sciencedirect.com/journal/academic-pathology/about/pathology-competencies-for-medical-education-pcme.^1^

## Secondary objective

Objective HWC2.4 Molecular Basis of Leukemia and Lymphoma. Describe how understanding the molecular pathogenesis of leukemia and lymphoma can suggest targets for therapeutic intervention and give examples of diseases currently treated by targeted therapy.

Competency 2: Organ System Pathology; Topic: Hematopathology: White Cell Disorders, Lymph Nodes, Spleen, and Thymus (HWC); Learning Goal 2: Genetic Mutations in Hematologic Malignancy.

## Patient presentation

A 63-year-old man presents to his primary care physician with 3 months of worsening fatigue, abdominal pain, and fullness. He has not had a fever, chills, or weight loss, but does note having had multiple respiratory infections over the past 6 months, most recently pneumonia, which resolved 6 weeks earlier. He does not have any personal or family history of cancer, autoimmune disease, or hematologic illness. The patient has never used tobacco products or illicit drugs, nor is he taking any medications. After completing a comprehensive history, no other pertinent findings are identified.

## Diagnostic findings, Part 1

On physical exam, vital signs are as follows: heart rate is 80 beats per minute, respiratory rate is 12 breaths per minute with 98% oxygen saturation, blood pressure is 123/81 mm Hg, and body temperature is 37°C. Physical exam reveals splenomegaly, conjunctival pallor, and multiple bruises on his arms and shins in various stages of healing. The pulmonary, cardiac, and neurologic exams are without abnormalities and there are no palpable lymph nodes.

## Questions/discussion points, Part 1

### Based on the patient presentation, what is the differential diagnosis, and what additional information would be most helpful next?

Fatigue is a common patient concern and carries a broad differential; however, the physical exam finding of conjunctival pallor raises concerns for anemia. The patient presents with abdominal pain with fullness, and a physical exam demonstrates splenomegaly. Taken together, these findings can help narrow the differential. Causes of splenomegaly include infection, malignancy, hematologic disorders, and liver disease. Recurrent respiratory infections in an adult–in the absence of environmental, toxin, or infectious exposures; anatomic abnormalities; or history of risk factors for respiratory infections, such as asthma–is concerning for immunodeficiencies. These may include white blood cell disorders, malignancies, or an acquired immunodeficiency syndrome. Given repeated concerns for a hematologic origin, the best next step would be to obtain a complete blood count and differential.

## Diagnostic findings, Part 2

Based on the clinical presentation, a complete blood count (CBC) and automated differential count are ordered. The results are provided in Table 1 and Table 2, respectively.

**Table 1:** Complete blood count.

Parameter	Patient	Reference range
WBC	2.1 K/mm^3^	4–10.4 K/mm^3^
RBC	3.8 M/mm^3^	4.36–5.78 M/mm^3^
Hemoglobin	11.5 g/dL	13.8–17.3 g/dL
Hematocrit	35.50%	39.5–50.2 %
MCV	92 fL	81-95 fL
Platelets	77 K/mm^3^	141–377 K/mm^3^

WBC: white blood cell; RBC: red blood cell; MCV: mean corpuscular volume.

**Table 2:** Automated differential count.

Parameter	Patient	Reference range
% Neutrophils	42%	Not reported
% Lymphocytes	55%	Not reported
% Monocytes	1%	Not reported
% Eosinophils	2%	Not reported
ABS Neutrophils	0.88 K/mm^3^	2.20–8.85 K/mm^3^
ABS Lymphocytes	1.15 K/mm^3^	1.09–3.30 K/mm^3^
ABS Monocytes	0.02 K/mm^3^	0.10–0.80 K/mm^3^
ABS Eosinophils	0.04 K/mm^3^	0.30–0.61 K/mm^3^

ABS: absolute.

## Questions/discussion points, Part 2

### How do you interpret the laboratory values in Table 1, Table 2?

The CBC demonstrates pancytopenia, a decrease in all three blood cell lineages: red blood cells (RBCs), white blood cells (WBCs), and platelets. The decreased hemoglobin and hematocrit with a normal mean corpuscular volume (MCV) is indicative of normocytic anemia. Next, from Table 1, the WBC is 2.1K/mm^3^ and is below the given reference range, indicating leukopenia. Table 2 is an automated differential, a breakdown of the WBC types into both percentages and absolute values. The absolute count for neutrophils (0.88K/mm^3^) and monocytes (0.02K/mm^3^) is also below the reference ranges, indicating a neutropenia and monocytopenia. Finally, the platelet count (<141K/mm^3^) is below the reference range, indicating thrombocytopenia.

### What is the differential diagnosis based on the clinical presentation and CBC thus far?

Pancytopenia carries a broad differential, including both neoplastic and non-neoplastic conditions and diagnostic considerations are dependent on patient age, underlying conditions, medications, and physical examination. In adults, benign causes include nutritional deficiencies, medication effect, toxic exposure, infection, autoimmune diseases, peripheral destruction/consumption, and splenic sequestration (splenomegaly), among others. Neoplastic conditions include primary bone marrow neoplasms such as acute or chronic leukemias, myelodysplastic syndrome, multiple myeloma, and lymphoma, as well as metastatic cancer infiltrating and effacing the bone marrow space.^2^, ^2^, b.

In addition to a comprehensive history and physical exam, utilizing the WBC count and differential can help narrow such a broad differential. First, consider benign entities. Leukocytes will increase and decrease in various benign conditions; for example, infection may cause a rise in overall WBC count with the WBC differential reflecting the infectious pathogen. Other infections, however, such as HIV, may result in a low WBC count. Patients using medications with known agranulocytosis, those with a rheumatologic disorder, or those with nutritional deficiencies may have a reduced absolute neutrophil count. Eosinophilia may be the result of allergic disorders, asthma, or a hypersensitivity reaction. It is important to consider the benign conditions when working through a WBC count and differential, but also keep hematologic neoplasms in mind. Low counts in multiple types of white blood cells and/or a significant elevation in one type raise suspicions for a bone marrow process. Given the patient's current presentation of fatigue, recurrent infections, splenomegaly, and CBC findings showing multiple cytopenias, the most likely explanation is a hematologic disorder or other bone marrow infiltrative disorder.

### How does the WBC count and differential narrow the differential diagnosis? What would be the next step in the diagnostic evaluation?

Neutrophils are the most abundant type of WBC made in the bone marrow. Disruptions in bone marrow function, such as those caused by acute leukemia or other bone marrow infiltrative disorders, can impair hematopoiesis, reducing the production and maturation of neutrophils and other WBCs. This impacts their distribution into the peripheral blood, reflected in the CBC and differential as neutropenia and other cytopenias.

In contrast, chronic leukemias such as chronic myeloid leukemia (CML) and chronic lymphocytic leukemia (CLL) are associated with leukocytosis, an increased WBC count. In these cases, the neoplastic cells retain their ability to mature and enter the peripheral blood in large quantities. The distinction between CML and CLL depends on the type of cells seen on the differential (maturing neutrophils in CML versus lymphocytes in CLL).^3^ An exception is hairy cell leukemia, a chronic leukemia of lymphocytic origin that commonly presents with leukopenia rather than leukocytosis. Finally, lymphoma remains a consideration, but the lack of lymphadenopathy or B-symptoms (fever, night sweats, and weight loss) makes lymphoma less likely than leukemia in this case.

The significant monocytopenia is a key feature of this patient's WBC differential and can be associated with myelofibrosis, aplastic anemia, glucocorticoid use, sepsis, hemodialysis, and hairy cell leukemia.^4^ Since there are still multiple hematologic disorders on our differential, further studies would be appropriate. Because obtaining a bone marrow biopsy is a more invasive procedure, the best next step would be to review a peripheral blood smear, which may give us a clue about the underlying bone marrow pathology.

### How does a peripheral blood smear review help narrow down the differential diagnosis for this patient?

**Fig. 1:**
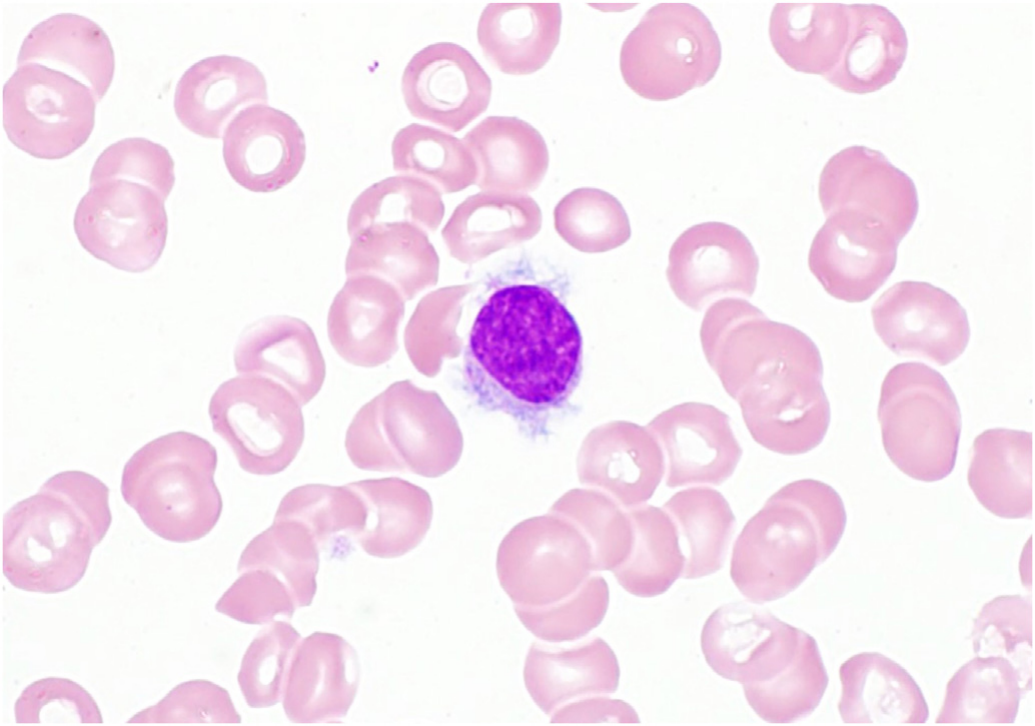
Peripheral smear. Note the lymphocyte with the abundant cytoplasm and hairy projections. (Wright-Giemsa, 1000x).

Review of the peripheral blood smear confirms the pancytopenia: neutrophils, monocytes, RBCs, and platelets are decreased in number but without morphologic abnormalities. There is no increased polychromasia of the RBCs and no evidence of hemolysis (no spherocytes or schistocytes). Also identified on the peripheral smear are rare, scattered abnormal lymphocytes that are generally small to intermediate in size with round nuclear contours, clumped chromatin, and a variable amount of cytoplasm characterized by hairy projections that are distributed uniformly around the circumference of the cell (Fig. 1). Notably, no blasts are seen on the peripheral smear, making acute leukemia less likely. Overall, these features are most compatible with involvement by a mature lymphoid process.

In a patient with a clinical history of fatigue, recurrent infections, and physical exam findings of splenomegaly and evidence of pancytopenia, the identification of these rare abnormal lymphocytes–particularly in conjunction with monocytopenia–warrants a high suspicion that this patient has hairy cell leukemia.

## Diagnostic findings, Part 3

**Fig. 2:**
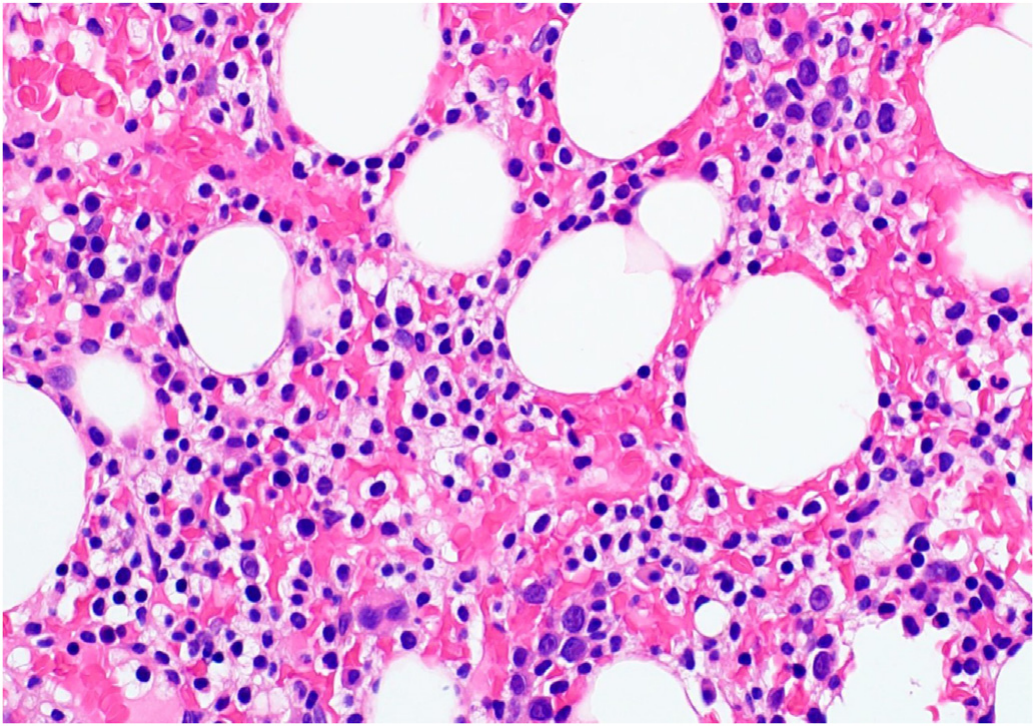
Bone marrow biopsy. Hematoxylin and eosin (400x). Note the classic “fried egg” appearance is typical for hairy cell leukemia.

A bone marrow biopsy is performed and submitted for evaluation and workup. Images from the bone marrow biopsy are provided in Fig. 2.

## Questions/discussion points, Part 3

### What would you expect to see on the bone marrow biopsy?

Hairy cell leukemia (HCL) has a variety of infiltrative patterns, ranging from inconspicuous patches to solid sheets of cells that replace the normal marrow.^5^ The classic appearance of a hairy cell on histologic examination is the so-called “fried-egg” morphology: a round to oval nucleus surrounded by an abundance of clear cytoplasm (Fig. 2). Reticulin fibrosis is commonly seen in the bone marrow due to the fibronectin released by the hairy cells, which may impact the ability to obtain aspirates from the bone marrow and result in a “dry tap.” The amount of reticulin often corresponds to the amount of hairy cell infiltrate present in the bone marrow.^5^^,^^6^

### What is the classic immunohistochemical and flow cytometric profile of hairy cell leukemia?

**Fig. 3:**
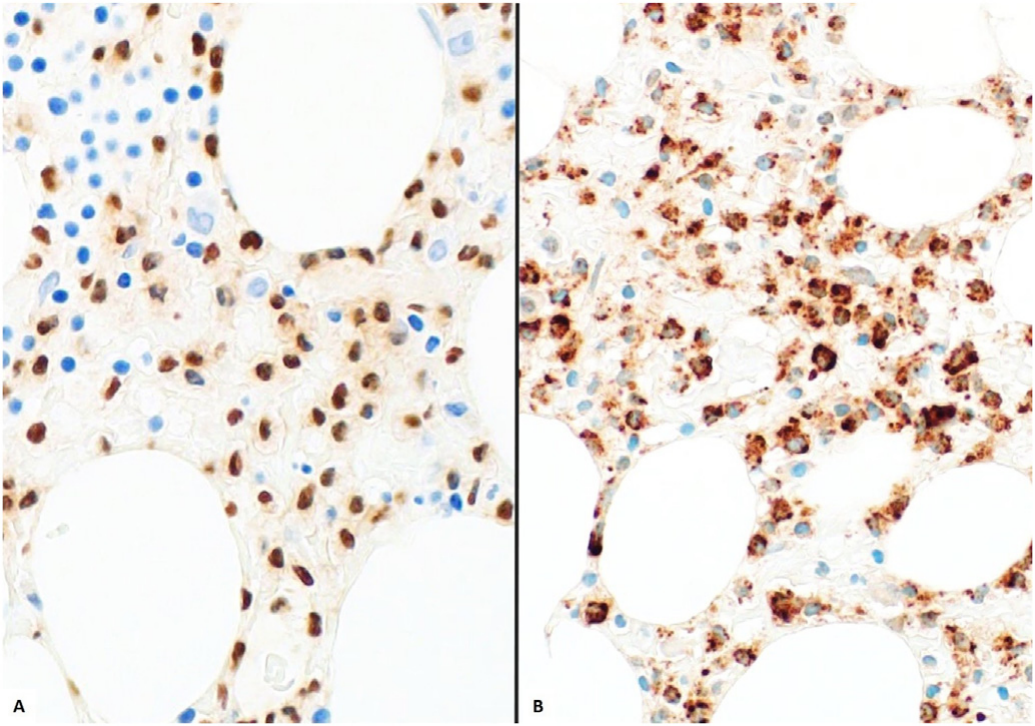
Neoplastic B-cells are immunoreactive for A) PAX5 (400x) and B) TRAP (400x). TRAP: tartrate-resistant acid phosphatase.

Due to the variability and often inconspicuous appearance of HCL in the bone marrow, immunohistochemical (IHC) stains can be used to help support this diagnosis. Hairy cell leukemia is a neoplasm of mature B-lymphocytes, making markers such as CD20 and PAX5 effective for highlighting the infiltrate.^4^^,^^5^ It is important to note that CD20 expression may be diminished or entirely lost in patients receiving rituximab, an anti-CD20 drug.^3^ T-bet and TRAP (tartrate-resistant acid phosphatase) are stains that are strongly positive in HCL, but may also be weakly positive in other B-cell lymphoproliferative disorders (Fig. 3).^6^ DBA44 acts on a surface membrane protein and is positive in a variety of B-cell lymphoproliferative disorders, but it can help highlight the classic hairy projections.^5^^,^^7^ Annexin 1 is very specific for HCL, but its cross-reactivity with myeloid cells can make its use limited to diffusely infiltrative disease.^4^^,^^5^^,^^7^

**Fig. 4:**
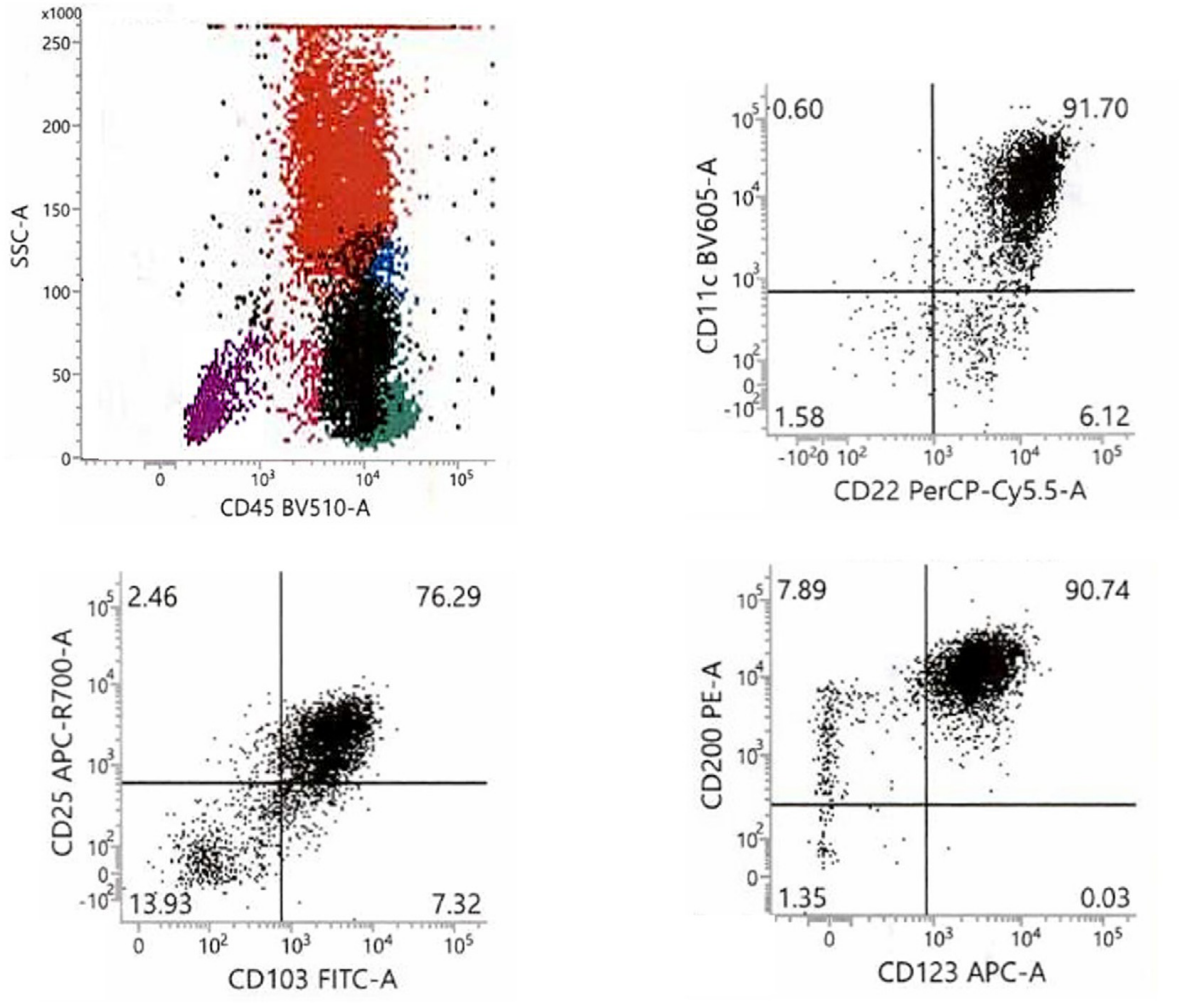
Flow cytometry. Hairy cells (the Black population) have characteristic positivity for CD22, CD103, CD123, and CD45.

Hairy cell leukemia has a characteristic immunophenotype by flow cytometry, which can solidify the diagnosis. This characteristic pattern includes expression of CD11c, CD22 (bright), CD25, CD45, CD103, C123, CD200 (bright), and bright (strongly positive) surface immunoglobulin (Fig. 4). Hairy cell leukemia cells are generally negative for CD5, CD10, and CD23.^4^^,^^5^ Notably, HCL cells are often higher on side light scatter criteria (often overlapping with monocytes) due to their abundant cytoplasm.

### What molecular genetic alteration is seen in most cases of hairy cell leukemia, and what role does it play in the underlying pathogenesis of disease?

Nearly all cases of HCL harbor the *BRAF* V600E mutation.^8^^,^^9^ This *BRAF* V600E mutation is responsible for constitutively activating the RAS-RAF-MEK-ERK pathway, which ultimately leads to cell cycle progression and cell survival.^9^^,^^10^ While this mutation can be seen in a variety of cancers, it is rarely observed in other mature B-cell lymphoproliferative disorders.^8^^,^^9^ The IHC stain, VE1, a *BRAF* V600E antibody, can identify cells positive for the mutation, making it a great tool for distinguishing HCL from other B-cell lymphoproliferative disorders and mimickers. Molecular methods of detection can also be used, including standalone polymerase chain reaction (PCR)-based assays and as part of a larger multi-gene panel using next-generation sequencing (NGS).

### How is hairy cell leukemia treated, and what is the clinical course?

Hairy cell leukemia is an indolent B-cell lymphoproliferative disorder with a 10-year overall survival rate of 92.4%, credited to advancements in therapy.^10^ Initial therapy for symptomatic patients is with a purine nucleoside analog, such as cladribine and pentostatin, with or without the addition of the anti-CD20 drug, rituximab.^4^ CD20 is a B-cell membrane protein and has become a target for treatment in a variety of non-Hodgkin lymphomas such as CLL, and multiple autoimmune conditions. Relapses of HCL are relatively common and tend to occur many years later; up to 50% of patients relapse by 10 years.^10^ Patients who have relapsed may opt to restart a purine analog with rituximab, or they may be considered for a newer treatment. Because the molecular pathogenesis of HCL includes a *BRAF* V600E mutation, a BRAF inhibitor, vemurafenib, has become a targeted treatment option.^4^ HCL patients are followed regularly with peripheral counts, and the reemergence of cytopenias is an indication for a repeat bone marrow biopsy to assess for relapsed disease.^4^

## Teaching points

• CBCs and differentials should be interpreted in the context of the patient's clinical presentation and medical history. These findings can guide the differential diagnosis and the need for additional studies.
• CBCs and differentials are compared to the reference ranges to identify abnormalities such as low or high counts, which can also help narrow the differential diagnosis and guide additional studies such as a peripheral smear review, bone marrow biopsy, flow cytometry, and molecular testing.
• Using an immunophenotypic profile derived from IHC stains and flow cytometry, in conjunction with results of cytogenetic and molecular testing, clinicians can identify specific abnormalities, which can guide the treatment selection.
• HCL is an indolent B-cell lymphoproliferative disorder involving the bone marrow, peripheral blood, and spleen. Neoplastic cells show circumferential projections on peripheral smear and a “fried egg” appearance on histologic sections.
• HCL typically affects middle-age-to-older adults and presents with fatigue, recurrent infections, easy bruising or bleeding, and splenomegaly. A CBC with differential on these patients often reveals pancytopenia with a profound monocytopenia.
• The classic immunophenotypic profile of HCL includes IHC positivity for CD20, TRAP, and Annexin 1. Flow cytometric analysis demonstrates positivity for CD11c, CD22 (bright), CD25, CD103, C123, and CD200 (bright).
• The vast majority of HCL patients have a *BRAF* V600E mutation, which leads to constitutive activation of pathways responsible for cell proliferation and survival.
• Treatment for HCL includes purine analogs, anti-CD20 drugs, and BRAF inhibitors. Late relapses are common, and regular follow-up is recommended.

## Funding

The article processing fee for this article was funded by an Open Access Award given by the Society of ‘67, which supports the mission of the Association for Academic Pathology to produce the next generation of outstanding investigators and educational scholars in the field of pathology. This award helps to promote the publication of high-quality original scholarship in *Academic Pathology* by authors at an early stage of academic development.

## Declaration of competing interest

The authors declare that there are no competing interests.
